# Retracted: Difference between Mechanical Alignment in Navigation and Scanogram during Total Knee Arthroplasty

**DOI:** 10.1155/2020/8934138

**Published:** 2020-07-09

**Authors:** Advances in Orthopedics

At the request of the authors, the article titled “Difference between Mechanical Alignment in Navigation and Scanogram during Total Knee Arthroplasty” [1] is being retracted.

During discussion among the authors following publication, it was found that one of the authors had mistakenly entered data into an incorrect location in a spreadsheet. The dataset cannot be corrected because it is not possible to identify which data entries are incorrect, and therefore, the article is being retracted at the request of the authors due to these concerns.
